# Uncertainty-aware prediction of chemical reaction yields with graph neural networks

**DOI:** 10.1186/s13321-021-00579-z

**Published:** 2022-01-10

**Authors:** Youngchun Kwon, Dongseon Lee, Youn-Suk Choi, Seokho Kang

**Affiliations:** 1 Samsung Advanced Institute of Technology, Samsung Electronics Co. Ltd., 130 Samsung-ro, Yeongtong-gu, Suwon, Republic of Korea; 2 Department of Computer Science and Engineering, Seoul National University, 1 Gwanak-ro, Gwanak-gu, Seoul, Republic of Korea; 3 Department of Industrial Engineering, Sungkyunkwan University, 2066 Seobu-ro, Jangan-gu, Suwon, Republic of Korea

**Keywords:** Chemical reaction yield prediction, Uncertainty-aware prediction, Graph neural network, Deep learning

## Abstract

In this paper, we present a data-driven method for the uncertainty-aware prediction of chemical reaction yields. The reactants and products in a chemical reaction are represented as a set of molecular graphs. The predictive distribution of the yield is modeled as a graph neural network that directly processes a set of graphs with permutation invariance. Uncertainty-aware learning and inference are applied to the model to make accurate predictions and to evaluate their uncertainty. We demonstrate the effectiveness of the proposed method on benchmark datasets with various settings. Compared to the existing methods, the proposed method improves the prediction and uncertainty quantification performance in most settings.

## Introduction

In organic chemistry, the prediction of chemical reaction yields is an important research topic in chemical synthesis planning [1, 2]. This enables the estimation of the overall yield of a complex synthetic pathway and the detection of low-yield reactions that negatively affect the overall yield. It also provides clues for designing new reactions that provide higher yields to save on the time and cost required for experimental syntheses.

Machine learning has achieved remarkable success in the data-driven prediction of chemical reaction yields [1, 3–7]. The main concept is to construct a prediction model that predicts the yield of a chemical reaction by learning from previously accumulated data comprising a number of chemical reactions annotated with their experimentally measured yields. The successful application of a prediction model enables fast and efficient estimation of chemical reaction yields without performing experimental syntheses, which are costly and time-consuming.

Early studies represented each chemical reaction as a fixed-size vector of handcrafted features, such as molecular fingerprints and chemical property descriptors, and constructed an off-the-shelf prediction model on top of the vector representation [3–5, 8]. The limitation of this approach is that the choice of adequate features relies on chemical knowledge and intuition, and some inherent information to the original reaction may be lost in the representation. With advances in deep learning [9], recent studies have applied deep neural networks constructed on a more informative representation of a chemical reaction. Schwaller et al. [6, 10] used simplified molecular-input line-entry system (SMILES) to represent a chemical reaction. To predict the reaction yield, they fine-tuned a bidirectional encoder representations from transformers (BERT) model pre-trained using a reaction SMILES database [11] to predict the yield. Saebi et al. [7] represented a chemical reaction as a set of graphs, on which a graph neural network was constructed to predict the yield.

In this paper, we present an alternative method for predicting chemical reaction yields. As a prediction model, we adapt a graph neural network that directly operates on the graph representation of a chemical reaction in a permutation-invariant fashion. We use uncertainty-aware learning and inference in the model to make accurate predictions of yields and determine the confidence of predictions.

## Methods

### Data representation

We suppose that a chemical reaction consists of a number of reactants and a single product. This chemical reaction is labeled with its reaction yield. Each instance is represented as $( \mathcal {R},\mathcal {P}, y )$, where $\mathcal {R} = \{\mathcal {G}^{R,1},\ldots ,\mathcal {G}^{R,m}\}$ and $\mathcal {P} = \{\mathcal {G}^{P}\}$ are the set of *m* reactants and the resulting product in the reaction, respectively, and *y* is the reaction yield. The number of reactants *m* can be different for each reaction.

**Fig. 1:**
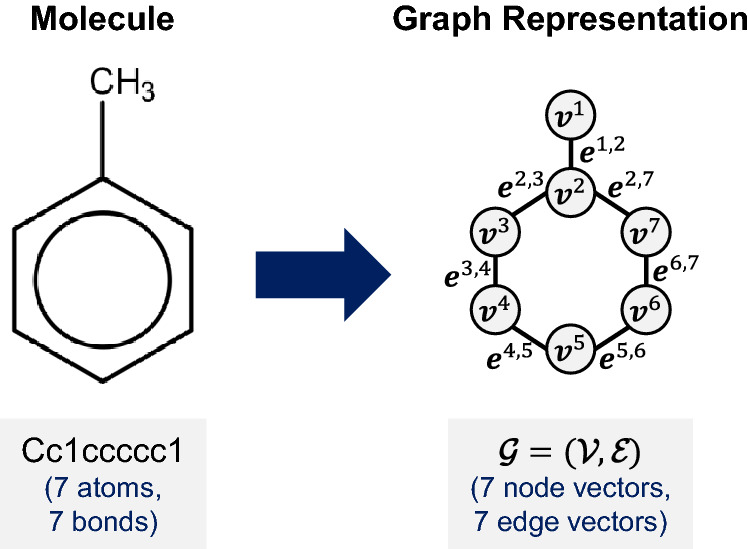
Illustrative example of the graph representation for a molecule

Each molecule in $\mathcal {R}$ and $\mathcal {P}$ is defined as an undirected graph $\mathcal {G}=(\mathcal {V},\mathcal {E})$, where $\mathcal {V}$ and $\mathcal {E}$ represent the set of nodes and the set of edges, respectively. The node feature vectors $\mathbf {v}^j \in \mathcal {V}$ and edge feature vectors $\mathbf {e}^{j,k} \in \mathcal {E}$ are associated with heavy atoms (e.g., C, N, O, and F) and their bonds (e.g., single, double, triple, and aromatic), respectively. Hydrogen atoms are treated implicitly. The number of heavy atoms and bonds in each molecule is the same as the number of node feature vectors and edge feature vectors in the corresponding graph representation, respectively. Figure 1 illustrates an example of the graph representation of a molecule.

For the *j*-th atom, $\mathbf {v}^j=(v^{j,1},\ldots ,v^{j,p})$ is a vector indicating the atom type, formal charge, degree, hybridization, number of hydrogens, valence, chirality, whether it accepts or donates electrons, whether it is aromatic, whether it is in a ring, and associated ring sizes. For the bond between the *j*-th and *k*-th atoms, $\mathbf {e}^{j,k}=(e^{j,k,1},\ldots ,e^{j,k,q})$ is a vector indicating the bond type, stereochemistry, whether it is in a ring, and whether it is conjugated.

### Prediction model

**Fig. 2:**
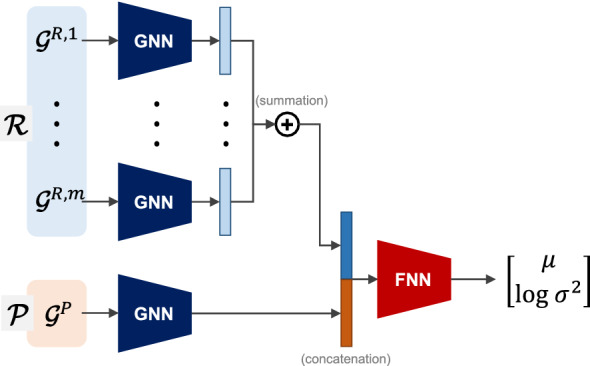
Architecture of the prediction model

To predict the reaction yield *y*, we introduce a predictive distribution for *y* conditioned on the set of reactants $\mathcal {R}$ and product $\mathcal {P}$, denoted by $p_\theta (y|\mathcal {R},\mathcal {P})$, which is modeled as a normal distribution as follows:

1 $$\begin{aligned} p_\theta (y|\mathcal {R},\mathcal {P}) =\mathcal {N}(y|\mu ,\sigma ^2), \end{aligned}$$

where $\mu$ and $\sigma ^2$ are the mean and variance of the distribution, respectively. We parameterize the predictive distribution $p_\theta$ using a neural network *f* that produces $\mu$ and $\sigma ^2$ as a function of $\mathcal {R}$ and $\mathcal {P}$ with a set of parameters $\theta$:

2 $$\begin{aligned} (\mu , \sigma ^2) = f(\mathcal {R}, \mathcal {P};\theta ). \end{aligned}$$

To construct the neural network *f*, we adapt the architecture presented by Saebi *et al.* [7] to process two sets of molecular graphs with advanced neural network modules. Figure 2 illustrates the architecture used in this study. The architectural details of each component are presented next.

A message passing neural network (MPNN) [12] is used as the GNN component of *f* to process each molecular graph $\mathcal {G}$ in $\mathcal {R}$ and $\mathcal {P}$. The GNN is designed to take $\mathcal {G}$ as the input and return the graph representation vector $\mathbf {r}$ as the output:

3 $$\begin{aligned} \mathbf {r} = \text {GNN}(\mathcal {G}). \end{aligned}$$

In the GNN, we apply multiple message passing steps using an edge network as a message function and a gated recurrent unit (GRU) network as an update function to generate node representation vectors. We then apply a set2set model [13] as a readout function for global pooling over the node representation vectors to obtain a graph-level embedding that is invariant to the order of the nodes. The embedding is sparsified by a fully-connected layer to obtain the graph representation vector $\mathbf {r}$. The use of the GNN renders the representation invariant to graph isomorphism.

We summate the graph representation vectors for $\mathcal {R} = \{\mathcal {G}^{R,1},\ldots ,\mathcal {G}^{R,m}\}$. This makes the representation invariant with respect to the order of the reactants. The summated vector is concatenated with the graph representation vector $\mathcal {P} = \{\mathcal {G}^{P}\}$ to generate the reaction representation vector $\mathbf {h}$:

4 $$\begin{aligned} \mathbf {h} = \left[ \sum _{l=1}^{m} {\mathbf {r}^{R,l}}, {\mathbf {r}^{P}} \right] . \end{aligned}$$

The reaction representation vector $\mathbf {h}$ is further processed by a feed-forward neural network (FNN) with two output units. The first unit returns the predictive mean $\mu$. The second unit returns the log predictive variance $\log \sigma ^2$.

The main advantages of the prediction model *f* presented in this study can be summarized as follows. First, the input for the model is the graph representation of a chemical reaction, which can directly encompass various atom and bond features regarding their chemical properties that make the representation more informative. Second, the model can handle chemical reactions of varying sizes with different numbers of reactants as the input. Third, the output of the model is invariant to permutations of reactants in the input reaction and is also invariant to permutations of atoms in each of the reactants/products. Fourth, the output of the model specifies the corresponding predictive distribution, which allows for uncertainty-aware learning and inference.

### Uncertainty-aware learning

The learning procedure aims to train the prediction model *f* such that it can estimate the predictive mean $\mu$ and variance $\sigma ^2$ of the unknown yield *y* for a chemical reaction $(\mathcal {R}, \mathcal {P})$. For the model *f* to learn from data, we construct a training dataset of *N* chemical reactions and their yields, denoted by $\mathcal {D}=\{(\mathcal {R}_i, \mathcal {P}_i, y_i)\}_{i=1}^N$.

We train the model *f* based on the maximum likelihood estimation. Based on the normality assumption for the predictive distribution $p_\theta$, the log-likelihood is given by:

5 $$\begin{aligned} \begin{aligned} \log p_\theta (y|\mathcal {R},\mathcal {P})&= -\frac{1}{2}\log (2\pi \sigma ^2 ) - \frac{1}{2} \frac{(y-\mu )^2}{\sigma ^2} \\&= -\frac{1}{2} \log (2\pi ) - \frac{1}{2} \left[ \frac{(y -\mu ) ^2}{\sigma ^2} + \log \sigma ^2 \right] . \end{aligned} \end{aligned}$$

Given a training dataset $\mathcal {D}$, the model is trained to minimize the objective function $\mathcal {J}$:

6 $$\begin{aligned} \mathcal {J}(\theta ) = &(1-\lambda ) \cdot \frac{1}{N} \sum _{i=1}^{N} (y_i -\mu _i) ^2 \\&+ \lambda \cdot \frac{1}{N} \sum _{i=1}^{N} \left[ \frac{(y_i -\mu _i) ^2}{\sigma _i^2} + \log \sigma _i^2 \right] , \end{aligned}$$

which involves two learning objectives with the hyperparameter $\lambda$ that controls the relative strength of each objective. The first term is to minimize the conventional mean squared error over the training dataset $\mathcal {D}$, which corresponds to the maximization of the log-likelihood over $\mathcal {D}$ under the homoscedasticity assumption. The second term is to maximize the log-likelihood over $\mathcal {D}$ under the heteroscedasticity assumption. The first term contributes to stabilizing the training with respect to the predictive mean $\mu$. The second term enables the predictive variance $\sigma ^2$ to quantify the aleatoric uncertainty caused by the inherent noise in $\mathcal {D}$.

### Uncertainty-aware inference

Once trained, the prediction model *f* is used to predict the yields of new chemical reactions. We employ the Monte-Carlo (MC) dropout [14] for the Bayesian approximation of the model *f*. Following the Bayesian approach, the approximate predictive distribution *q* is given by

7 $$\begin{aligned} q(y_*|\mathcal {R}_*,\mathcal {P}_*) = \int p_\theta (y_*|\mathcal {R}_*,\mathcal {P}_*) d\theta . \end{aligned}$$

Given a query reaction $(\mathcal {R}_*,\mathcal {P}_*)$, we wish to predict the unknown yield ${y}_*$ of the reaction as well as to quantify the uncertainty of the prediction. We empirically derive the MC estimates by sampling *T* predictions $\{(\hat{\mu }_*^{(t)}, \hat{\sigma }_*^{2(t)})\}_{t=1}^T$ based on stochastic forward passes through the model *f* with dropout applied. Because some hidden units are randomly dropped out at each forward pass, the *T* predictions vary for the same reaction. The variability in the predictions is primarily caused by the epistemic uncertainty of the model *f* owing to the insufficiency of the training dataset $\mathcal {D}$.

For prediction, the predictive mean can be estimated by averaging over $\{\hat{\mu }_*^{(t)}\}_{t=1}^T$:

8 $$\begin{aligned} \text {E}_{q(y_*|\mathcal {R}_*,\mathcal {P}_*)}[y_*] \simeq \frac{1}{T} \sum _{t=1}^T \hat{\mu }_*^{(t)}. \end{aligned}$$

This is used as the prediction of ${y}_*$.

For uncertainty quantification, the predictive variance can be estimated as:

9 $$\begin{aligned} \text {Var}_{q(y_*|\mathcal {R}_*,\mathcal {P}_*)}[{y}_*] \simeq \frac{1}{T} \sum _{t=1}^T \hat{\sigma }_*^{2 (t)} + \frac{1}{T} \sum _{t=1}^T \left( {\hat{\mu }_*^{(t)}} - \bar{\mu }_* \right) ^2, \end{aligned}$$

where $\bar{\mu }_* = \frac{1}{T} \sum _{t=1}^T \hat{\mu }_*^{(t)}$. This is used as the uncertainty score for the prediction. The predictive variance can be decomposed into two types of uncertainty [15]. The first term corresponds to the aleatoric uncertainty, which accounts for the statistical uncertainty caused by inherent noise in the dataset $\mathcal {D}$. The second term corresponds to the epistemic uncertainty, which accounts for the systemic uncertainty in the model *f* caused by the insufficiency of $\mathcal {D}$.

The prediction of chemical reaction yields supports the identification of high-yield reactions from a pool of possible candidates in an efficient manner. The prerequisite is that the prediction model must be as accurate as possible. In practice, the prediction model may be imperfect and result in inaccurate predictions. To overcome this issue, we can selectively use the model based on uncertainty quantification. Because a high prediction uncertainty tends to cause erroneous predictions, the rejection of uncertain predictions would be beneficial for the actual use of the prediction model. If the prediction uncertainty is sufficiently low, we can use the model with confidence to identify whether a reaction has a high yield. Otherwise, the model abstains from predicting. Rejected cases can be carefully investigated by chemists in terms of their yields.

**Table 1:** Description of benchmark datasets

Dataset	No. reactions	No. reactants per reaction	No. products per reaction
Buchwald-Hartwig	3955	6	1
Suzuki-Miyaura	5760	6–14	1

### Experimental investigation

#### Datasets

We investigate the effectiveness of the proposed method using the following two benchmark datasets: Buchwald-Hartwig [3] and Suzuki-Miyaura [16]. In these datasets, each reaction was annotated with a measured yield ranging from 0% to 100%. The summary statistics of the datasets are presented in Table 1.

The Buchwald-Hartwig dataset was released by Ahneman et al. [3]. They conducted high-throughput experiments on the class of Pd-catalyzed Buchwald-Hartwig C-N cross-coupling reactions. They experimented on combinations of 15 aryl halides, 4 ligands, 3 bases, and 23 additives. A total of 3955 reactions were reported with their measured yields. The studies [3–6] evaluated the performance of the chemical reaction yield prediction on this dataset.

The Suzuki-Miyaura dataset was released by Perera et al. [16]. They conducted high-throughput experiments on the class of Suzuki-Miyaura cross-coupling reactions. 15 couplings of electrophiles and nucleophiles across combinations of 12 ligands, 8 bases, and 4 solvents were considered, resulting in measured yields for a total of 5760 reactions. The studies [6, 16, 17] have investigated this dataset.

For experimental investigations, we use 10 random shuffles for each benchmark dataset and 4 out-of-sample splits of the Buchwald-Hartwig dataset [3, 6].

#### Implementation

In the experimental investigation, we use the following configurations for the proposed method. For the GNN component of the model, the node representation vectors and graph representation vectors have dimensions of 64 and 1024, respectively. The graph representation vectors were set to have higher dimensionality because they are summated over multiple reactants to obtain the reaction representation vector. The number of message passing steps and set2set processing steps are both set to 3. Increasing the size of the GNN component may provide better performance, but it also incurs higher computational costs and memory usage. Thus, we set it to moderately large so that it can be trained in a reasonable time. The FNN component of the model has two fully-connected layers with 512 dimensions, followed by an output layer. During training, we standardize the yield *y* to have a mean of 0 and a variance of 1 over the training dataset $\mathcal {D}$. A dropout rate of 0.1 is applied to the fully-connected layers in the FNN component. The hyperparameter $\lambda$ in the objective function $\mathcal {J}$ is set to 0.1. L2 regularization with a factor of $10^{-5}$ is applied to the parameters $\theta$. To train the model *f*, we update the parameters $\theta$ for 500 epochs using the Adam optimizer with a batch size of 128. The learning rate is set to $10^{-3}$ for the initial epochs and decayed to $10^{-4}$ and $10^{-5}$ over the last 100 epochs. We did not consider hyperparameter optimization through holdout validation, because it is unsuitable when the training dataset is very small. At inference, we set the number of forward passes *T* to 30 for MC dropout. We use Equation 8 and Equation 9 for the prediction and uncertainty score, respectively.

The proposed method is implemented using PyTorch in Python. The source code used in this study is available online at http://github.com/seokhokang/reaction_yield_nn/. The results of the experimental investigations are reported and discussed in the following section.

## Results and discussion

### Prediction and uncertainty quantification

We investigated the effectiveness of the proposed method for predicting the chemical reaction yields on the Buchwald-Hartwig and Suzuki-Miyaura datasets. For the proposed method, we derived two ablations by adjusting the hyperparameter $\lambda$ in the objective function $\mathcal {J}$. For the first ablation, the model was trained using only homoscedastic loss by setting $\lambda =0$, which is equivalent to fixing the predictive variance $\sigma$ to 1. For the second ablation, the model was trained using only heteroscedastic loss by setting $\lambda =1$. For baselines, we considered YieldBERT [6] and YieldBERT-DA [10], which demonstrated superior performance compared to the other methods presented in the literature [3–5]. YieldBERT adapted a pre-trained BERT encoder [11] to predict the chemical reaction yield as a function of the reaction SMILES. YieldBERT-DA is an extension of YieldBERT based on data augmentation, which increases the quantity of the training dataset using SMILES randomization. For YieldBERT-DA, the prediction uncertainty score was computed using the prediction variance obtained from the test-time augmentation, as implemented in [10]. The source codes for YieldBERT and YieldBERT-DA are available online at https://github.com/rxn4chemistry/rxn_yields/, which we used to reproduce the experimental results. Consequently, a total of five methods were compared: YieldBERT, YieldBERT-DA, and the proposed method with $\lambda =0$, 1, and 0.1.

For performance evaluation, we split each dataset into training and test sets. We then trained the prediction model using the training set and evaluated its performance on the test set. To examine the effects of training set size on performance, the training/test splits were varied as 70/30, 50/50, 30/70, 20/80, 10/90, 5/95, and 2.5/97.5. Regarding prediction performance, we used the following three measures calculated on the test set: mean absolute error (MAE), root mean squared error (RMSE), and coefficient of determination (R$^2$). Uncertainty quantification performance was evaluated in terms of the Spearman rank correlation coefficient $\rho$ between the absolute prediction error and uncertainty score on the test set [10, 18].

**Table 2:** Comparison of prediction and uncertainty quantification performance on benchmark datasets

Dataset	Training/test split	Measure	YieldBERT	YieldBERT-DA	Proposed		
					$\lambda = 0$	$\lambda = 1$	$\lambda = 0.1$
Buchwald-Hartwig	70/30	MAE (%p)	3.990 ± 0.153	3.090 ± 0.118	3.009 ± 0.045	2.953 ± 0.058	$\mathbf{2.920} \pm \mathbf{0.056}$
		RMSE (%p)	6.014 ± 0.272	4.799 ± 0.261	4.509 ± 0.116	4.535 ± 0.136	$\mathbf{4.433} \pm \mathbf{0.085}$
		R$^2$	0.951 ± 0.005	0.969 ± 0.004	0.973 ± 0.002	0.972 ± 0.002	$\mathbf{0.974} \pm \mathbf{0.001}$
		Spearman $\rho$	–	0.439 ± 0.037	0.254 ± 0.027	$\mathbf{0.445} \pm \mathbf{0.020}$	0.421 ± 0.031
	50/50	MAE (%p)	4.792 ± 0.124	3.744 ± 0.150	3.614 ± 0.095	$\mathbf{3.482} \pm \mathbf{0.107}$	3.497 ± 0.090
		RMSE (%p)	7.288 ± 0.198	5.877 ± 0.348	5.484 ± 0.193	5.481 ± 0.355	$\mathbf{5.387} \pm \mathbf{0.202}$
		R$^2$	0.928 ± 0.004	0.953 ± 0.006	0.959 ± 0.003	0.959 ± 0.005	$\mathbf{0.961} \pm \mathbf{0.003}$
		Spearman $\rho$	–	$\mathbf{0.460} \pm \mathbf{0.021}$	0.227 ± 0.021	0.419 ± 0.020	0.401 ± 0.014
	30/70	MAE (%p)	6.075 ± 0.222	4.833 ± 0.167	4.677 ± 0.174	$\mathbf{4.463} \pm \mathbf{0.150}$	4.483 ± 0.165
		RMSE (%p)	9.338 ± 0.424	7.822 ± 0.463	7.227 ± 0.407	7.053 ± 0.439	$\mathbf{6.970} \pm \mathbf{0.403}$
		R$^2$	0.882 ± 0.011	0.917 ± 0.010	0.929 ± 0.008	0.933 ± 0.009	$\mathbf{0.934} \pm \mathbf{0.008}$
		Spearman $\rho$	–	$\mathbf{0.464} \pm \mathbf{0.020}$	0.229 ± 0.035	0.407 ± 0.022	0.385 ± 0.029
	20/80	MAE (%p)	6.862 ± 0.212	5.781 ± 0.252	5.605 ± 0.236	5.319 ± 0.179	$\mathbf{5.311} \pm \mathbf{0.154}$
		RMSE (%p)	10.306 ± 0.303	9.164 ± 0.668	8.567 ± 0.472	8.357 ± 0.400	$\mathbf{8.204} \pm \mathbf{0.372}$
		R$^2$	0.857 ± 0.008	0.886 ± 0.017	0.901 ± 0.011	0.906 ± 0.009	$\mathbf{0.909} \pm \mathbf{0.008}$
		Spearman $\rho$	–	$\mathbf{0.457} \pm \mathbf{0.017}$	0.208 ± 0.044	0.373 ± 0.040	0.343 ± 0.029
	10/90	MAE (%p)	8.607 ± 0.387	7.705 ± 0.236	7.605 ± 0.420	7.244 ± 0.229	$\mathbf{7.196} \pm \mathbf{0.274}$
		RMSE (%p)	12.393 ± 0.499	11.633 ± 0.293	11.468 ± 0.699	11.002 ± 0.436	$\mathbf{10.875} \pm \mathbf{0.448}$
		R$^2$	0.793 ± 0.016	0.818 ± 0.009	0.822 ± 0.022	0.837 ± 0.013	$\mathbf{0.841} \pm \mathbf{0.013}$
		Spearman $\rho$	–	$\mathbf{0.432} \pm \mathbf{0.024}$	0.148 ± 0.036	0.384 ± 0.040	0.345 ± 0.031
	5/95	MAE (%p)	12.117 ± 0.789	$\mathbf{9.651} \pm \mathbf{0.338}$	10.056 ± 0.501	10.609 ± 1.610	9.677 ± 0.408
		RMSE (%p)	16.740 ± 0.950	14.073 ± 0.687	14.636 ± 0.672	14.693 ± 1.467	$\mathbf{14.041} \pm \mathbf{0.492}$
		R$^2$	0.622 ± 0.042	0.733 ± 0.027	0.711 ± 0.026	0.707 ± 0.063	$\mathbf{0.734} \pm \mathbf{0.019}$
		Spearman $\rho$	–	$\mathbf{0.411} \pm \mathbf{0.024}$	0.002 ± 0.058	0.398 ± 0.141	0.399 ± 0.058
	2.5/97.5	MAE (%p)	15.979 ± 0.817	12.243 ± 0.631	12.409 ± 0.558	13.508 ± 2.745	$\mathbf{11.747} \pm \mathbf{1.005}$
		RMSE (%p)	20.463 ± 0.623	17.151 ± 0.677	17.384 ± 0.775	17.992 ± 2.530	$\mathbf{16.586} \pm \mathbf{1.364}$
		R$^2$	0.436 ± 0.034	0.604 ± 0.031	0.593 ± 0.037	0.556 ± 0.130	$\mathbf{0.628} \pm \mathbf{0.062}$
		Spearman $\rho$	–	$\mathbf{0.381} \pm \mathbf{0.038}$	0.016 ± 0.067	0.309 ± 0.176	0.300 ± 0.075
Suzuki-Miyaura	70/30	MAE (%p)	8.128 ± 0.344	6.598 ± 0.270	6.233 ± 0.207	6.118 ± 0.212	$\mathbf{6.116} \pm \mathbf{0.223}$
		RMSE (%p)	12.073 ± 0.463	10.524 ± 0.482	9.522 ± 0.454	9.495 ± 0.430	$\mathbf{9.467} \pm \mathbf{0.459}$
		R$^2$	0.815 ± 0.013	0.859 ± 0.012	0.885 ± 0.010	0.885 ± 0.009	$\mathbf{0.886} \pm \mathbf{0.010}$
		Spearman $\rho$	–	$\mathbf{0.439} \pm \mathbf{0.018}$	0.324 ± 0.026	0.432 ± 0.024	0.425 ± 0.026
	50/50	MAE (%p)	8.922 ± 0.235	7.539 ± 0.153	6.872 ± 0.089	$\mathbf{6.702} \pm \mathbf{0.082}$	6.725 ± 0.089
		RMSE (%p)	13.148 ± 0.270	11.797 ± 0.250	10.272 ± 0.138	$\mathbf{10.225} \pm \mathbf{0.128}$	$\mathbf{10.225} \pm \mathbf{0.135}$
		R$^2$	0.780±0.009	0.823 ± 0.007	0.866 ± 0.003	$\mathbf{0.867} \pm \mathbf{0.003}$	$\mathbf{0.867} \pm \mathbf{0.003}$
		Spearman $\rho$	–	$\mathbf{0.439} \pm \mathbf{0.019}$	0.322 ± 0.021	0.432 ± 0.017	0.430 ± 0.012
	30/70	MAE (%p)	10.094 ± 0.346	8.804 ± 0.249	8.021 ± 0.094	$\mathbf{7.740} \pm \mathbf{0.109}$	7.847 ± 0.094
		RMSE (%p)	14.614 ± 0.381	13.337 ± 0.357	11.726 ± 0.152	$\mathbf{11.526} \pm \mathbf{0.166}$	11.593 ± 0.136
		R$^2$	0.729 ± 0.014	0.774 ± 0.012	0.825 ± 0.004	$\mathbf{0.831} \pm \mathbf{0.005}$	0.829 ± 0.004
		Spearman $\rho$	–	$\mathbf{0.432} \pm \mathbf{0.018}$	0.292 ± 0.012	0.428 ± 0.013	0.417 ± 0.008
	20/80	MAE (%p)	11.229 ± 0.247	10.017 ± 0.338	9.147 ± 0.185	$\mathbf{8.726} \pm \mathbf{0.172}$	8.793 ± 0.191
		RMSE (%p)	15.966 ± 0.381	14.851 ± 0.576	13.115 ± 0.298	12.754 ± 0.316	$\mathbf{12.734} \pm \mathbf{0.347}$
		R$^2$	0.676 ± 0.015	0.719 ± 0.022	0.781 ± 0.010	0.793 ± 0.010	$\mathbf{0.794} \pm \mathbf{0.011}$
		Spearman $\rho$	–	$\mathbf{0.432} \pm \mathbf{0.014}$	0.274 ± 0.020	0.429 ± 0.017	0.408 ± 0.018
	10/90	MAE (%p)	13.528 ± 0.395	11.954 ± 0.443	11.439 ± 0.185	$\mathbf{10.625} \pm \mathbf{0.249}$	10.739 ± 0.211
		RMSE (%p)	18.734 ± 0.530	17.129 ± 0.683	15.967 ± 0.326	$\mathbf{15.097} \pm \mathbf{0.421}$	15.164 ± 0.344
		R$^2$	0.554 ± 0.025	0.627 ± 0.030	0.676 ± 0.013	$\mathbf{0.711} \pm \mathbf{0.016}$	0.708 ± 0.013
		Spearman $\rho$	–	0.389 ± 0.022	0.221 ± 0.027	$\mathbf{0.390} \pm \mathbf{0.019}$	0.382 ± 0.019
	5/95	MAE (%p)	15.695 ± 0.618	14.294 ± 0.507	14.214 ± 0.504	$\mathbf{13.364} \pm \mathbf{0.223}$	13.451 ± 0.353
		RMSE (%p)	21.181 ± 0.724	20.016 ± 0.661	19.421 ± 0.588	$\mathbf{18.463} \pm \mathbf{0.308}$	18.511 ± 0.392
		R$^2$	0.430 ± 0.040	0.491 ± 0.034	0.521 ± 0.029	$\mathbf{0.567} \pm \mathbf{0.014}$	0.565 ± 0.018
		Spearman $\rho$	–	0.355 ± 0.026	0.144 ± 0.052	$\mathbf{0.389} \pm \mathbf{0.045}$	0.330 ± 0.034
	2.5/97.5	MAE (%p)	17.666 ± 0.496	17.587 ± 0.690	18.061 ± 0.571	$\mathbf{16.705} \pm \mathbf{1.090}$	17.189 ± 0.813
		RMSE (%p)	22.967 ± 0.804	23.780 ± 0.793	24.121 ± 0.655	$\mathbf{22.156} \pm \mathbf{1.273}$	22.943 ± 0.887
		R$^2$	0.330 ± 0.047	0.282 ± 0.047	0.261 ± 0.039	$\mathbf{0.375} \pm \mathbf{0.072}$	0.331 ± 0.051
		Spearman $\rho$	–	$\mathbf{0.291} \pm \mathbf{0.025}$	0.028 ± 0.054	0.280 ± 0.074	0.223 ± 0.081

Table 2 reports the average and standard deviation of the results over the 10 repetitions. In terms of prediction performance, the proposed method outperformed all the baseline methods. Although YieldBERT-DA was the best baseline method, the MAE and RMSE values of the proposed method reduced by around 5$\sim$10% compared to those of YieldBERT-DA on both benchmark datasets. The higher prediction performance indicates that the proposed method can provide more accurate predictions of yields for new reactions. Regarding uncertainty quantification performance, the proposed method yielded a Spearman $\rho$ comparable to that of YieldBERT-DA.

For the proposed method, the prediction performance with $\lambda = 1$ was slightly better than that with $\lambda = 0$. The uncertainty quantification performance with $\lambda = 1$ was far better than that with $\lambda = 0$, which implies that capturing the aleatoric uncertainty is beneficial. Compared to the ablations, setting $\lambda = 0.1$ yielded a better trade-off between prediction performance and uncertainty quantification performance. The results demonstrated that the use of both homoscedastic and heteroscedastic losses helped to improve performance.

### Out-of-sample prediction

We also evaluated the performance of the proposed method for out-of-sample prediction. As in [6, 10], we used four out-of-sample training/test splits of the Buchwald-Hartwig dataset, which we denote by Test 1, Test 2, Test 3, and Test 4. In each split, certain additives are absent from the training set but only appear in the test set. The proposed method was compared with YieldBERT and YieldBERT-DA. The training configurations and evaluation scheme were the same as before. The experiments were repeated five times independently using different random seeds.

Table 3 summarizes the results averaged over the five repetitions. Overall, the proposed method was comparable to the best of the baseline methods for out-of-sample prediction. In terms of prediction performance, the proposed method performed best on Test 2 and Test 4, while was comparable or inferior to the best baseline on Test 1 and Test 3. Among the baselines, YieldBERT-DA yielded a lower performance than YieldBERT on average. For uncertainty quantification performance, the proposed method yielded the highest Spearman $\rho$ for Test 1, Test 3, and Test 4.

**Table 3:** Comparison of prediction and uncertainty quantification performance on out-of-sample splits of Buchwald-Hartwig dataset

Out-of-sample split	Measure	YieldBERT	YieldBERT-DA	Proposed ($\lambda = 0.1$)
Test 1	MAE (%p)	7.351 ± 0.099	$\mathbf{7.015} \pm \mathbf{0.758}$	8.082 ± 0.827
	RMSE (%p)	$\mathbf{11.441} \pm \mathbf{0.342}$	11.761 ± 1.398	13.746 ± 1.175
	R$^2$	$\mathbf{0.824} \pm \mathbf{0.010}$	0.811 ± 0.047	0.744 ± 0.042
	Spearman $\rho$	–	0.380 ± 0.065	$\mathbf{0.454} \pm \mathbf{0.046}$
Test 2	MAE (%p)	7.266 ± 0.724	6.588 ± 0.328	$\mathbf{6.300} \pm \mathbf{0.647}$
	RMSE (%p)	11.144 ± 1.267	9.886 ± 0.741	$\mathbf{9.476} \pm \mathbf{1.027}$
	R$^2$	0.829 ± 0.037	0.866 ± 0.020	$\mathbf{0.876} \pm \mathbf{0.026}$
	Spearman $\rho$	–	$\mathbf{0.494} \pm \mathbf{0.044}$	0.397 ± 0.043
Test 3	MAE (%p)	9.129 ± 0.745	11.052 ± 0.950	$\mathbf{8.986} \pm \mathbf{0.314}$
	RMSE (%p)	$\mathbf{14.276} \pm \mathbf{0.820}$	18.041 ± 1.395	14.939 ± 0.622
	R$^2$	$\mathbf{0.741} \pm \mathbf{0.030}$	0.585 ± 0.067	0.717 ± 0.024
	Spearman $\rho$	–	0.406 ± 0.065	$\mathbf{0.423} \pm \mathbf{0.031}$
Test 4	MAE (%p)	13.671 ± 1.067	18.422 ± 0.620	$\mathbf{13.190} \pm \mathbf{0.754}$
	RMSE (%p)	19.679 ± 1.397	24.279 ± 0.494	$\mathbf{18.774} \pm \mathbf{0.566}$
	R$^2$	0.444 ± 0.077	0.157 ± 0.034	$\mathbf{0.496} \pm \mathbf{0.031}$
	Spearman $\rho$	–	0.366 ± 0.100	$\mathbf{0.461} \pm \mathbf{0.040}$

### Selective prediction with rejection

We investigated the effectiveness of the proposed method for selective prediction using 70/30 splits of benchmark datasets. For the proposed method, prediction uncertainty was quantified using the total predictive variance in Eq. 9. Because it can be decomposed into aleatoric and epistemic uncertainties, we conducted an ablation study to examine the effects of each component. The first ablation quantified the prediction uncertainty using the aleatoric uncertainty term. The second ablation used the epistemic uncertainty term. The proposed method was compared to the best baseline method, YieldBERT-DA, for which the uncertainty quantification was based on the test-time augmentation.

To evaluate the selective prediction performance, we rejected the prediction for a reaction if its uncertainty score was above a certain threshold. The threshold controls the trade-off between prediction accuracy and coverage. As performance measures, we computed the MAE and RMSE on the test set with various prediction coverage rates ranging from 100% to 30%.

**Fig. 3:**
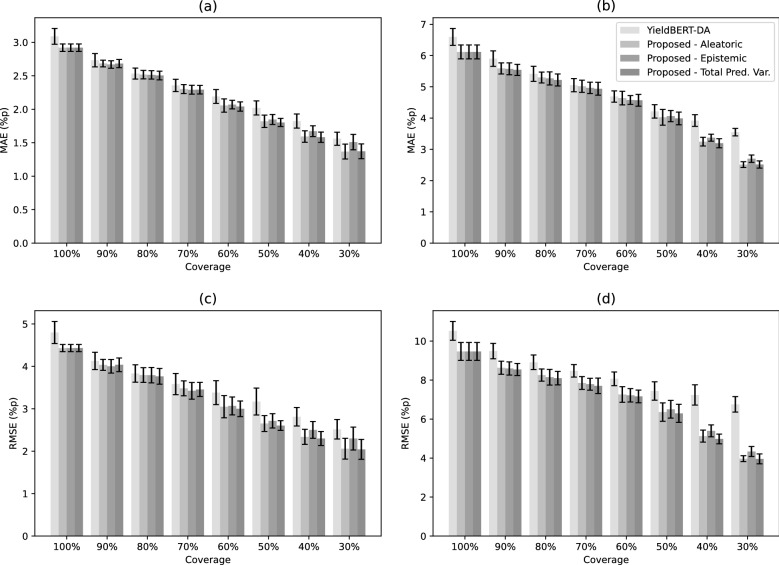
Summary for comparison of selective prediction performance on benchmark datasets: **a** MAE (%p) on Buchwald-Hartwig; **b** MAE (%p) on Suziki-Miyaura; **c** RMSE (%p) on Buchwald-Hartwig; **d** RMSE (%p) on Suziki-Miyaura

Tables 4 and 5 present the comparison results for the selective prediction performance in terms of the MAE and RMSE with various prediction coverage rates, which are summarized in Fig. 3. The results clearly demonstrated that a high uncertainty score for a reaction causes its predicted yield to be less accurate for all compared methods. Reducing the prediction coverage with more rejections led to a significant improvement in the prediction performance. The proposed method outperformed YieldBERT-DA in most cases. The MAE and RMSE decreased by over 10% and were nearly halved at 90% and 40% coverages, respectively, for both datasets.

Regarding the two ablations of the proposed method, the selective prediction performance with the epistemic uncertainty was superior at higher prediction coverages, whereas that with the aleatoric uncertainty was better at lower coverages. Compared to the ablations, using the total predictive variance combining the aleatoric and epistemic uncertainty improved the performance by taking their individual strengths to detect erroneous predictions.

**Table 4:** Comparison of selective prediction performance in terms of MAE (%p)

Dataset	Coverage	YieldBERT-DA	Proposed ($\lambda = 0.1$)		
			Aleatoric	Epistemic	Total Pred. Var.
Buchwald-Hartwig	100%	3.090 ± 0.118	$\mathbf{2.920} \pm \mathbf{0.056}$	$\mathbf{2.920} \pm \mathbf{0.056}$	$\mathbf{2.920} \pm \mathbf{0.056}$
	90%	2.733 ± 0.099	2.684 ± 0.050	$\mathbf{2.669} \pm \mathbf{0.056}$	2.683 ± 0.061
	80%	2.534 ± 0.082	2.518 ± 0.064	2.514 ± 0.063	$\mathbf{2.505} \pm \mathbf{0.065}$
	70%	2.357 ± 0.092	2.302 ± 0.067	$\mathbf{2.292} \pm \mathbf{0.067}$	2.293 ± 0.064
	60%	2.191 ± 0.103	2.056 ± 0.099	2.070 ± 0.064	$\mathbf{2.041} \pm \mathbf{0.069}$
	50%	2.020 ± 0.105	1.820 ± 0.093	1.847 ± 0.075	$\mathbf{1.803} \pm \mathbf{0.061}$
	40%	1.824 ± 0.106	1.593 ± 0.086	1.672 ± 0.081	$\mathbf{1.582} \pm \mathbf{0.077}$
	30%	1.560 ± 0.098	$\mathbf{1.368} \pm \mathbf{0.112}$	1.509 ± 0.115	1.372 ± 0.111
Suzuki-Miyaura	100%	6.598 ± 0.270	$\mathbf{6.116} \pm \mathbf{0.223}$	$\mathbf{6.116} \pm \mathbf{0.223}$	$\mathbf{6.116} \pm \mathbf{0.223}$
	90%	5.902 ± 0.247	5.589 ± 0.178	5.575 ± 0.191	$\mathbf{5.542} \pm \mathbf{0.178}$
	80%	5.415 ± 0.242	5.298 ± 0.174	5.269 ± 0.210	$\mathbf{5.219} \pm \mathbf{0.192}$
	70%	5.052 ± 0.211	5.018 ± 0.196	4.966 ± 0.183	$\mathbf{4.939} \pm \mathbf{0.208}$
	60%	4.690 ± 0.181	4.641 ± 0.218	4.579 ± 0.140	$\mathbf{4.570} \pm \mathbf{0.188}$
	50%	4.213 ± 0.214	4.025 ± 0.252	4.064 ± 0.179	$\mathbf{3.989} \pm \mathbf{0.203}$
	40%	3.921 ± 0.188	3.245 ± 0.140	3.372 ± 0.111	$\mathbf{3.195} \pm \mathbf{0.145}$
	30%	3.549 ± 0.120	$\mathbf{2.510} \pm \mathbf{0.093}$	2.701 ± 0.118	2.514 ± 0.115

**Table 5:** Comparison of selective prediction performance in terms of RMSE (%p)

Dataset	Coverage	YieldBERT-DA	Proposed ($\lambda =0.1$)		
			Aleatoric	Epistemic	Total Pred. Var.
Buchwald-Hartwig	100%	4.799 ± 0.261	$\mathbf{4.433} \pm \mathbf{0.085}$	$\mathbf{4.433} \pm \mathbf{0.085}$	$\mathbf{4.433} \pm \mathbf{0.085}$
	90%	4.129 ± 0.205	4.036 ± 0.130	$\mathbf{4.003} \pm \mathbf{0.160}$	4.037 ± 0.161
	80%	3.833 ± 0.206	3.796 ± 0.173	3.793 ± 0.182	$\mathbf{3.765} \pm \mathbf{0.185}$
	70%	3.583 ± 0.249	3.482 ± 0.176	$\mathbf{3.424} \pm \mathbf{0.196}$	3.456 ± 0.166
	60%	3.382 ± 0.282	3.050 ± 0.261	3.068 ± 0.211	$\mathbf{3.001} \pm \mathbf{0.184}$
	50%	3.171 ± 0.317	2.653 ± 0.187	2.716 ± 0.168	$\mathbf{2.605} \pm \mathbf{0.115}$
	40%	2.812 ± 0.218	2.338 ± 0.178	2.503 ± 0.197	$\mathbf{2.300} \pm \mathbf{0.166}$
	30%	2.518 ± 0.229	2.059 ± 0.245	2.299 ± 0.270	$\mathbf{2.044} \pm \mathbf{0.235}$
Suzuki-Miyaura	100%	10.524 ± 0.482	$\mathbf{9.467} \pm \mathbf{0.459}$	$\mathbf{9.467} \pm \mathbf{0.459}$	$\mathbf{9.467} \pm \mathbf{0.459}$
	90%	9.485 ± 0.395	8.632 ± 0.334	8.592 ± 0.338	$\mathbf{8.540} \pm \mathbf{0.310}$
	80%	8.911 ± 0.373	8.254 ± 0.314	8.146 ± 0.403	$\mathbf{8.098} \pm \mathbf{0.347}$
	70%	8.473 ± 0.323	7.848 ± 0.329	7.787 ± 0.305	$\mathbf{7.702} \pm \mathbf{0.397}$
	60%	8.063 ± 0.353	7.260 ± 0.400	7.218 ± 0.343	$\mathbf{7.160} \pm \mathbf{0.328}$
	50%	7.439 ± 0.470	6.357 ± 0.470	6.503 ± 0.456	$\mathbf{6.293} \pm \mathbf{0.466}$
	40%	7.236 ± 0.521	5.126 ± 0.306	5.394 ± 0.306	$\mathbf{4.980} \pm \mathbf{0.250}$
	30%	6.754 ± 0.398	3.968 ± 0.152	4.337 ± 0.257	**3.959 ± 0.252**

## Conclusion

We presented an uncertainty-aware method for predicting chemical reaction yields. We represented a chemical reaction as a set of graphs. We constructed a prediction model whose input was the graphs and output was the predictive mean and variance for the reaction yield. For a query reaction, the predictive mean of the model was used as the predicted yield and the predictive variance was used to quantify the uncertainty of the prediction, which allowed the model to avoid making predictions with high uncertainty. The effectiveness of the proposed method for chemical reaction yield prediction was successfully demonstrated through experimental validation on two benchmark datasets. We also demonstrated that a high predictive variance tends to cause a high prediction error, allowing for selective prediction with rejection.

The accurate prediction of chemical reaction yields with uncertainty quantification can assist in advanced synthesis planning considering imposed constraints in practice, including availability, variability, and budget limits. Future research directions for improving prediction performance will be to enrich the data representation of chemical reactions to make it more informative by incorporating various atom/bond features and molecular descriptors associated with reaction yields.

## Data Availability

The source code used in this study is available online at http://github.com/seokhokang/reaction_yield_nn/. The benchmark datasets are publicly accessible from https://github.com/rxn4chemistry/rxn_yields/.
